# Albumin Exchange in Alzheimer's Disease: Might CSF Be an Alternative Route to Plasma?

**DOI:** 10.3389/fneur.2019.01036

**Published:** 2019-10-18

**Authors:** Manuel Menendez-Gonzalez, Charles Gasparovic

**Affiliations:** ^1^Instituto de Investigación Sanitaria del Principado de Asturias, Oviedo, Spain; ^2^Department of Neurology, Hospital Universitario Central de Asturias, Oviedo, Spain; ^3^Department of Medicine, Universidad de Oviedo, Oviedo, Spain; ^4^The Mind Research Network, Albuquerque, NM, United States

**Keywords:** Alzheimer's disease, amyloid-beta-protein, CSF (cerebrospinal fluid), BBB (blood–brain barrier), therapy

## Abstract

Amyloid β (Aβ) in brain parenchyma is thought to play a central role in the pathogenesis of Alzheimer's disease (AD). Aβ is transported from the brain to the plasma via complex transport mechanisms at the blood-brain barrier (BBB). About 90–95% of plasma Aβ may be bound to albumin. Replacement of serum albumin in plasma has been proposed as a promising therapy for AD. However, the efficacy of this approach may be compromised by altered BBB Aβ receptors in AD, as well as multiple pools of Aβ from other organs in exchange with plasma Aβ, competing for albumin binding sites. The flow of interstitial fluid (ISF) into cerebrospinal fluid (CSF) is another major route of Aβ clearance. Though the concentration of albumin in CSF is much lower than in plasma, the mixing of CSF with ISF is not impeded by a highly selective barrier and, hence, Aβ in the two pools is in more direct exchange. Furthermore, unlike in plasma, Aβ in CSF is not in direct exchange with multiple organ sources of Aβ. Here we consider albumin replacement in CSF as an alternative method for therapeutic brain Aβ removal and describe the possible advantages and rationale supporting this hypothesis.

## Introduction

Aggregation of amyloid-β (Aβ) in the brain parenchyma and arterial walls and the formation of neurofibrillary tangles in neurons due to phosphorylated tau protein accumulation are the main histologic hallmarks of Alzheimer's disease (AD). While familial AD is associated with an increased production of Aβ_1−42_, the amyloid form most prone to aggregate, sporadic AD may be related to an imbalance between the production and the clearance of different forms of the Aβ peptide (1). Interstitial monomeric Aβ is in equilibrium with Aβ in oligomers and large insoluble fibrils (plaques) and cleared from the central nervous system (CNS) via at least three pathways: metabolic degradation, efflux across the blood–brain barrier (BBB) and efflux via perivascular and cerebrospinal fluid (CSF) routes. The relative importance of each of these pathways is under investigation (1, 2).

To cross the BBB, soluble Aβ in the interstitial fluid (ISF) of the brain's extracellular spaces must first diffuse to the capillary basement membrane, past pericytes and astrocytic endfeet, to reach the endothelium (3). Blockage of Aβ transcytosis across the endothelium accelerates the abnormal deposition of Aβ and is closely associated with neuronal degeneration (4–6). In addition to drainage via ISF to capillaries, Aβ reaches the CSF via perivascular spaces, where it can be cleared via CSF drainage routes (2). While the details and dynamics of these pathways are currently under investigation and the subject of much debate, the concentration of Aβ in CSF has been shown to be significantly altered in AD (7, 8).

Aβ is produced throughout the body and has a concentration in plasma and CSF of ~0.1–0.5 nM (9, 10). Its clearance relies on carrier proteins such as albumin, beta-2-macroglobulin, apolipoprotein E, apolipoprotein J (clusterin), low-density lipoprotein receptor-related protein-1 (LRP1), and transthyretin (11–15). A very small quantity of plasma proteins diffuse into CSF from plasma (e.g., albumin). Unlike plasma (~7 g protein/100 ml) or milky lymph (~2 g protein/100 ml), the CSF has only ~0.025 g protein/100 ml—mainly albumin. All albumin is synthesized in the liver and initially released into blood. Albumin is the most abundant protein in both plasma (~640 μM) and CSF (~3 μM) (16) and has been reported to be the primary carrier of Aβ in blood (17). However, there is also evidence that a soluble cleavage product of LRP1 (sLRP) may be the major plasma carrier of Aβ (15). In addition to Aβ, albumin also transports a variety of other endogenous and exogenous molecules and is involved in regulating oncotic pressure.

Some kinetic studies have supported a 1:1 stoichiometry for the binding of monomeric Aβ to albumin with a dissociation constant (K_d_) of ~5–10 μM (18–20), while others have shown that albumin preferentially binds oligomeric Aβ at 3 independent binding sites with much lower K_d_'s, in the range of 1–100 nM (21). A recent study has also supported a lower K_d_ for monomeric Aβ than previously reported (e.g., 180 nM for monomeric Aβ_1−42_) (22). Regardless of the study, these K_d_ values would explain the high percentage of binding of Aβ to albumin in blood (~90%) (17). In CSF, based on an assumed K_d_ of 5 μM, it has been estimated that ~40% of Aβ is bound to albumin (20). However, if the lower Kd's reported for Aβ binding to albumin are more accurate, then a much higher fraction of Aβ could be bound to CSF albumin. For example, if the effective CSF albumin-Aβ K_d_ is as low as 180 nM, as measured by Litus et al. for Aβ_1−42_, and assuming 1:1 monomeric binding without interference from other ligands, the percentage of albumin-bound Aβ in CSF could be as high as 94%.

Whether by direct binding of monomeric forms of Aβ or indirectly via oligomer binding and inhibition of further monomer addition, albumin also attenuates the growth of Aβ fibrils (20, 21, 23–25). The binding of cholesterol and fatty acids to albumin have been shown to weaken the binding of Aβ (22, 26), possibly contributing to the association of high dietary levels of these molecules with AD (26).

Changes in albumin are independently associated with aging and neurodegeneration. Low serum albumin is associated with increased odds of cognitive impairment in the elderly (27), and both plasma and CSF albumin oxidation are higher in AD patients compared to healthy controls (28). In a study on sheep, an increase in CSF albumin concentration in older sheep was attributed to a reduced production of CSF by the choroid plexus, even though the latter was observed to increase in size with age (29).

There is preliminary evidence from both animal and human studies that replacing plasma albumin using interventions such as hemodialysis and plasmapheresis may be effective in treating AD (30–33). In an ongoing clinical trial (34), the safety and efficacy of using plasma exchange to remove albumin-bound Aβ for treating AD is being explored (35–37). The concept behind this approach is that a reduction of the albumin-bound Aβ pool in plasma will, in turn, reduce Aβ levels in the parenchyma of the brain, known as the “peripheral sink” hypothesis (38–40). However, equilibrium between the brain and blood pools of Aβ is complex, involving distinct one-way receptor-mediated transport mechanisms on either side of the BBB, which have been shown to be altered in AD, thus reducing the net efflux of Aβ from the brain (2, 41). Moreover, Aβ bound to plasma albumin comes from several organ sources, not solely from the brain. These factors could potentially limit the additional efflux of Aβ from the brain after plasma albumin replacement.

While large insoluble Aβ fibrils forming the plaques of AD are the most conspicuous makers of the disease, a large and growing body of evidence supports that smaller soluble oligomers may be the most cytotoxic forms of Aβ. Soluble Aβ oligomers are capable of forming transmembrane ion channels (42, 43), disrupting homeostasis, and have been shown to have a number of cell surface and intracellular targets implicated in AD pathology, including components of synaptogenesis and neurofibrillary tangle formation (25, 44–46). Hence, trapping toxic oligomeric forms of Aβ is likely to be a major underlying mechanism for the therapeutic effect of albumin replacement on AD (47, 48).

Other possible therapeutic roles for albumin include its role as a transporter of the labile pool of Cu^2+^ ions in blood plasma (49). Dysregulation of Cu^2+^, leading to increases in oxidative stress and cellular damage, has been implicated in Aβ aggregation and fibril formation (50–52). Plasma exchange therapy may also serve to replace aged and glycated albumin. Both CSF and plasma proteins are involved in a vicious cycle of glycation during aging, as aging reduces CSF turnover and increases the exposure time of CSF proteins to glucose, resulting in more glycation of CSF proteins (53, 54). Protein properties are altered after glycation, resulting in a decreased degradation rate and a longer time to be eliminated (55). Glycated albumin, which has less positive charge than non-glycated albumin, more readily crosses brain barriers from the blood to the CSF (56). The increasing glycation of CSF proteins during aging may stimulate the formation and the consequent deposition of AGEs as well as oxidative stress in the brain (53).

In addition to plasma exchange, adding synthetic albumin to CSF has been explored as a more direct route to augmenting the capture and elimination of toxic forms of interstitial brain Aβ. In the 3xTg mouse model of AD, Ezra et al. showed that intraventricular infusion of synthetic serum albumin for 28 days, using an osmotic pump, led to a decrease of both Aβ monomers and oligomers in brain homogenates and amyloid plaques in histological samples (48). Additionally, performance in memory and fear conditioning tasks improved in treated animals and hyperphosphorylated tau was reduced and tubulin increased in brain homogenates, suggesting increased microtubule stability. Markers of BBB and myelin integrity were also assayed and demonstrated improvement in treated mice.

The positive findings of Ezra et al. on direct infusion of albumin into CSF along with the potential limitations of plasma exchange motivate us to propose an approach that combines aspects of both: CSF exchange with albumin replacement.

## The Therapeutic Hypothesis

**Figure 1:**
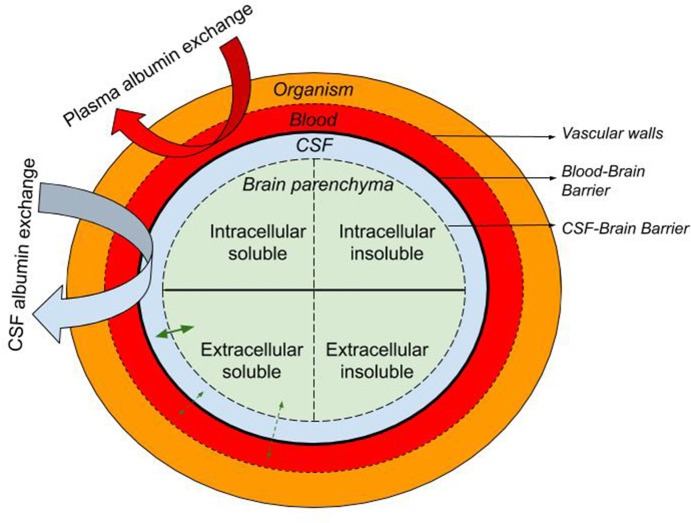
Schematic representation of the the blood, CSF, and brain intra- and extra-cellular pools of albumin-containing fluids with the organism. The equilibrium of soluble molecules (i.e., soluble Aβ) between the extracellular ISF, CSF, and plasma is represented with straight green arrows while curved arrows illustrate the CSF and plasma albumin exchange.

Here we describe the rationale supporting the therapeutic approach of exchanging endogenous albumin in CSF for synthetic albumin for the treatment of AD. Like plasma exchange, CSF exchange is a “peripheral sink” approach to affecting brain levels of Aβ, whereby Aβ in a pool that is in equilibrium with the brain pool of Aβ is replaced with Aβ-free fluid (Figure 1). This process is either continuous, as in dialysis, or repeated at intervals, as in plasma exchange. During or after the process, the Aβ-free pool re-establishes an equilibrium with the brain pool, drawing out extracellular brain Aβ as it does.

Though both plasma and CSF may act as “sinks” for interstitial Aβ, the drainage routes for either differ substantially. An early study on the relationship between plasma and CSF levels of Aβ in a transgenic mouse model of AD observed a significant and strong correlation between the levels in mice without detectable Aβ plaques but no significant correlation in mice after Aβ plaque formation (7). Subsequent studies have revealed that distinct and complex brain efflux and influx transport mechanisms are involved in the transfer of Aβ across the BBB (57, 58). Though the relationship between plasma and ISF levels is still not fully understood, in AD the Aβ transporter LRP1 on the abluminal side of the capillary endothelium has been reported to be reduced in number (41, 59) while the Aβ transporter receptor for advanced glycation end products (RAGE) on the luminal side of microvascular endothelium is increased (59). These alterations have been suggested to contribute to the net accumulation of interstitial Aβ in AD, favoring the formation of insoluble Aβ fibrils.

Given the possible impairment of the BBB Aβ transport mechanisms in AD, CSF exchange may create an Aβ sink with less impediments to drainage than plasma exchange, since there is no tight cellular barrier between the CSF and ISF compartments. A substantial fraction of the brain's metabolic waste and cellular/pathogen degradation debris is cleared by CSF/ISF drainage routes to lymphatic and venous outlets. Estimates for the elimination of Aβ via CSF have been as high as 25% (60) [reviewed by (61)]. The CSF that flows along perivascular spaces originates in the subarachnoid space and, by mechanisms still being explored, it may mix with ISF deep within the parenchyma, promoting the passage of cellular waste back to the CSF. Though the dynamics of these pathways, including that of the so-called “glymphatic” system (62), are still being debated, several studies have demonstrated the involvement of CSF drainage routes in normal Aβ clearance [reviewed by (61)].

The specific approach to CSF exchange we propose is a variation on liquorpheresis (cerebrospinal fluid filtration). Liquorpheresis is the process of filtering CSF in order to clear “toxic” molecules from the CSF (63). Though currently not in common use, it has been used to treat a number of neurological diseases and recent developmental efforts may foster its future use. Specifically, we propose to use liquorpheresis with albumin replacement to create a “CSF sink” of low Aβ within the brain. We hypothesize that this sink will pull the equilibrium between free and CSF albumin-bound Aβ in the brain more toward albumin-bound Aβ, as the latter is cleared from the brain either via the natural routes of CSF drainage or via the ongoing liquorpheresis filtering process itself.

## Discussion

Could CSF exchange be more efficacious than plasma exchange for the replacement of albumin to treat AD? We summarize three points from the discussion above that support the hypothesis that it could be:

1. The origin of CSF Aβ is mostly cerebral, whereas the origin of plasma Aβ is from the entire body, with just a small proportion coming from the brain. Hence, plasma exchange is non-specific for brain-derived Aβ and alters the homeostasis of Aβ across multiple organs. Indeed, plasma exchange has been shown to be followed by an initial overshoot of plasma Aβ (a “sawtooth” concentration pattern) (37), presumably as the multiple tissue-plasma equilibria are re-established. In contrast, CSF albumin replacement is a much more targeted therapeutic approach. Though the concentration of albumin is a factor of 20 less in CSF, it only binds Aβ that exists in the brain, even if some fraction of this Aβ derived from plasma via the BBB or choroid plexus.
2. If sLRP is the principal carrier of brain-derived Aβ as some studies suggest (15), then plasma albumin replacement may have only a minor effect on brain-derived Aβ, since the sLRP carrier is released from the luminal side of the BBB after LRP1-Aβ transcytosis and is hypothetically already tightly bound to Aβ. As acknowledged by Boada et al. in a preliminary report on clinical plasma exchange, its beneficial effects on AD may be due to factors not related to albumin-Aβ binding (37).
3. Even though the BBB may be the major route of interstitial Aβ clearance in healthy subjects, alterations in BBB Aβ transporters with age, leading to lower net Aβ efflux, may exacerbate or be a cause of AD. Adding to this potential limitation of plasma exchange is that Aβ efflux across the BBB is a saturable process (64, 65). Under these circumstances, the barriers to CSF-ISF exchange may present less of an impediment for Aβ clearance than the highly selective and transporter-dependent BBB barrier and CSF-ISF drainage routes might act as compensatory routes for clearing brain Aβ in AD (4).

There are several unknown factors that will impact the capacity of CSF exchange to enhance the clearance of brain Aβ. Most of these factors would be very challenging to model, given our still evolving knowledge of the normal routes and dynamics of CSF/ISF drainage, let alone the uncertainties regarding the *in vivo* binding kinetics of Aβ to albumin. Nonetheless, Ezra et al. (48) have shown that a relatively small addition of synthetic, Aβ-free albumin to intraventricular CSF, delivered via an osmotic pump, had measurable effects on Aβ plaque formation as well as several other markers of disease in a mouse model of AD. These results suggest similar studies on CSF albumin replacement via liquorpheresis to explore the possibility of achieving comparable effects with a relatively straightforward clinical tool that has been shown to be efficacious in treating other neurological diseases. Experimentation in animal models of AD will also be useful to compare the relative efficacies of liquorpheresis and plasma exchange to reduce brain Aβ levels.

Clinically, liquorpheresis may offer advantages in terms of safety over either CSF infusion by osmotic pump or plasma exchange. Infusing albumin either intrathecally or intraventricularly in humans poses the risk of increased intracranial pressure due to the osmotic properties of albumin, while equimolar albumin replacement during CSF exchange circumvents this possibility. Plasma exchange needs to be performed during long sessions in a hospital setting, with potential side effects associated with osmotic changes and the removal of other circulating factors, such as metabolites, cytokines, clotting factors, and hormones. Liquorpheresis, on the other hand, does not entail the removal of such factors and requires shorter periods of time since the CSF volume is much smaller than the blood volume. However, an advantage of plasma exchange is that it can be performed through a venous access while liquorpheresis requires a lumbar puncture. A comparison of the potential advantages and disadvantages plasma vs CSF exchange is given in Table 1.

**Table 1:** Comparison between plasma albumin exchange and CSF albumin exchange.

**Plasma albumin exchange**	**CSF albumin exchange**
The blood-sink mechanism of action relies on transport of Aβ through the BBB, which is a saturable process and is damaged in AD.	The CSF-sink mechanism of action relies on transport of Aβ through the CSF-brain barrier, circumventing BBB transport which is compromised in AD.
Many endogenous and pharmaceutical molecules can bind plasmatic albumin. Therefore, removing plasmatic albumin might interfere in many physiological functions and treatments.	The number of endogenous and pharmaceutical molecules binding CSF albumin is much lower than the number binding albumin in plasma. Therefore, removing CSF albumin would interfere less with normal physiologic function or other treatments.
Levels of albumin in plasma are much higher than in CSF. Therefore, the amount of albumin that needs to be removed in order to achieve a “sink effect” is also higher.	The amount of albumin in CSF is much lower than in plasma; therefore, the amount of albumin that needs to be removed in order to achieve a “sink effect” is relatively much lower.
Potential systemic side effects affecting multiple organs, including the CNS.	Potential side effects limited to the CNS.
Nonspecific for cerebral Aβ.	Specific for cerebral Aβ.
Plasma albumin exchange is a well-developed technique. • Infusion of albumin in plasma is a common clinical practice. • Devices for plasmapheresis are available in most hospitals. • Requires venous puncture.	• CSF albumin exchange would be a novel use of liquorpheresis. • Intrathecal infusion of albumin has never been attempted in humans. • Requires a lumbar puncture.

*Positive points are in green and negative points in red*.

## Conclusions

The accumulation of toxic oligomers of Aβ in the brain due to inadequate clearance from ISF may be a cause of AD. Reduction of Aβ in fluids peripheral to brain ISF may enhance Aβ clearance within the brain. Plasma exchange has been shown to be one promising approach to accomplish this in humans, as has intraventricular infusion of synthetic albumin in a mouse model of AD. Intrathecal CSF exchange (liquorpheresis) with albumin replacement may be an alternative approach that circumvents the potential disadvantages and clinical risks of either plasma exchange or CSF infusion. CSF exchange targets the pool of Aβ directly in exchange with brain ISF, without being impeded, as with Aβ transport via the BBB, by a highly selective, saturable, and possibly dysfunctional barrier. In terms of efficacy and safety, CSF exchange is a clinically tested therapeutic approach for the treatment of other neurological diseases and, unlike CSF infusion, does not risk disruption of osmotic homeostasis. Experimentation with animal models is needed to establish that CSF exchange performs as well as CSF infusion in reducing the hallmarks of AD, as well as to optimize its application. Additionally, such experiments could shed more light on Aβ dynamics in health and disease.

## Author Contributions

MM-G is the author of the therapeutic hypothesis. MM-G and CG wrote the manuscript.

### Conflict of Interest

The authors declare that the research was conducted in the absence of any commercial or financial relationships that could be construed as a potential conflict of interest.

## References

[1] Selkoe DJ Hardy J. The amyloid hypothesis of Alzheimer's disease at 25 years. EMBO Mol Med. (2016) 8:595–608. 10.15252/emmm.201606210 27025652 PMC4888851

[2] Tarasoff-Conway JM Carare RO Osorio RS Glodzik L Butler T Fieremans E. Clearance systems in the brain-implications for Alzheimer disease. Nat Rev Neurol. (2015) 11:457–70. 10.1038/nrneurol.2015.119 26195256 PMC4694579

[3] Sweeney MD Sagare AP Zlokovic BV. Blood-brain barrier breakdown in Alzheimer disease and other neurodegenerative disorders. Nat Rev Neurol. (2018) 14:133–50. 10.1038/nrneurol.2017.188 29377008 PMC5829048

[4] Simon MJ Iliff JJ. Regulation of cerebrospinal fluid (CSF) flow in neurodegenerative, neurovascular and neuroinflammatory disease. Biochim Biophys Acta. (2016) 1862:442–51. 10.1016/j.bbadis.2015.10.014 26499397 PMC4755861

[5] Weller RO Carare RO Boche D Amyloid: vascular and parenchymal. In: Reference Module in Neuroscience and Biobehavioral Psychology. (2017). 10.1016/B978-0-12-809324-5.02590-6

[6] Arbel-Ornath M Hudry E Eikermann-Haerter K Hou S Gregory JL Zhao L. Interstitial fluid drainage is impaired in ischemic stroke and Alzheimer's disease mouse models. Acta Neuropathol. (2013) 126:353–64. 10.1007/s00401-013-1145-2 23818064 PMC3810119

[7] DeMattos RB Bales KR Parsadanian M O'Dell MA Foss EM Paul SM. Plaque-associated disruption of CSF and plasma amyloid-β (Aβ) equilibrium in a mouse model of Alzheimer's disease. J Neurochem. (2002) 81:229–36. 10.1046/j.1471-4159.2002.00889.x 12064470

[8] Blennow K Hampel H Weiner M Zetterberg H. Cerebrospinal fluid and plasma biomarkers in Alzheimer disease. Nat Rev Neurol. (2010) 6:131–44. 10.1038/nrneurol.2010.4 20157306

[9] Lame ME Chambers EE Blatnik M Quantitation of amyloid beta peptides Aβ1–38, Aβ1–40, and Aβ1–42 in human cerebrospinal fluid by ultra-performance liquid chromatography–tandem mass spectrometry. Analyt Biochem. (2011) 419:133–9. 10.1016/j.ab.2011.08.010 21888888

[10] Seubert P Vigo-Pelfrey C Esch F Lee M Dovey H Davis D. Isolation and quantification of soluble Alzheimer's β-peptide from biological fluids. Nature. (1992) 359:325–7. 10.1038/359325a0 1406936

[11] Dergunov AD. Competition between serum amyloid protein and apoprotein E for binding with human serum albumin. Bull Exp Biol Med. (1992) 114:1791–3. 10.1007/BF00840464 1292684

[12] Liu Y-H Xiang Y Wang Y-R Jiao S-S Wang Q-H Bu X-L. Association between serum amyloid-beta and renal functions: implications for roles of kidney in amyloid-beta clearance. Mol Neurobiol. (2015) 52:115–9. 10.1007/s12035-014-8854-y 25119777

[13] Marr R Masliah E Amyloid-Beta Clearance in Alzheimer's Disease. Frontiers Media SA (2015). Available online at: https://books.google.com/books/about/Amyloid_beta_clearance_in_Alzheimer_s_di.html?hl=&id=qfdBCgAAQBAJ

[14] Yamamoto K Shimada H Ataka S Miki T Serum levels of albumin-beta-amyloid complexes are usable biomarkers for Alzheimer's disease. Alzheimer. Dement. (2013) 9:P233–4. 10.1016/j.jalz.2013.05.445

[15] Sagare A Deane R Bell RD Johnson B Hamm K Pendu R. Clearance of amyloid-β by circulating lipoprotein receptors. Nat Med. (2007) 13:1029–31. 10.1038/nm1635 17694066 PMC2936449

[16] Seyfert S Faulstich A Marx P. What determines the CSF concentrations of albumin and plasma-derived IgG? J Neurol Sci. (2004) 219:31–3. 10.1016/j.jns.2003.12.002 15050434

[17] Biere AL Ostaszewski B Stimson ER Hyman BT Maggio JE Selkoe DJ. Amyloid β-peptide is transported on lipoproteins and albumin in human plasma. J Biol Chem. (1996) 271:32916–22. 10.1074/jbc.271.51.32916 8955133

[18] Kuo YM Kokjohn TA Kalback W Luehrs D Galasko DR Chevallier N. Amyloid-beta peptides interact with plasma proteins and erythrocytes: implications for their quantitation in plasma. Biochem Biophys Res Commun. (2000) 268:750–6. 10.1006/bbrc.2000.2222 10679277

[19] Rózga M Kłoniecki M Jabłonowska A Dadlez M Bal W. The binding constant for amyloid Abeta40 peptide interaction with human serum albumin. Biochem Biophys Res Commun. (2007) 364:714–8. 10.1016/j.bbrc.2007.10.080 18028874

[20] Stanyon HF Viles JH. Human serum albumin can regulate amyloid-β peptide fiber growth in the brain interstitium: implications for Alzheimer disease. J Biol Chem. (2012) 287:28163–8. 10.1074/jbc.C112.360800 22718756 PMC3431649

[21] Milojevic J Melacini G. Stoichiometry and affinity of the human serum albumin-Alzheimer's Aβ peptide interactions. Biophys J. (2011) 100:183–92. 10.1016/j.bpj.2010.11.037 21190670 PMC3010178

[22] Litus EA Kazakov AS Sokolov AS Nemashkalova EL Galushko EI Dzhus UF. The binding of monomeric amyloid β peptide to serum albumin is affected by major plasma unsaturated fatty acids. Biochem Biophys Res Commun. (2019) 510:248–53. 10.1016/j.bbrc.2019.01.081 30685090

[23] Milojevic J Esposito V Das R Melacini G. Understanding the molecular basis for the inhibition of the Alzheimer's Aβ-peptide oligomerization by human serum albumin using saturation transfer difference and off-resonance relaxation NMR spectroscopy. J Am Chem Soc. (2007) 129:4282–90. 10.1021/ja067367+ 17367135

[24] Milojevic J Raditsis A Melacini G. Human serum albumin inhibits Aβ fibrillization through a “monomer-competitor” mechanism. Biophys J. (2009) 97:2585–94. 10.1016/j.bpj.2009.08.028 19883602 PMC2770600

[25] Domínguez-Prieto M Velasco A Vega L Tabernero A Medina JM. Aberrant co-localization of synaptic proteins promoted by Alzheimer's disease amyloid-β peptides: protective effect of human serum albumin. J Alzheimers Dis. (2017) 55:171–82. 10.3233/JAD-160346 27662292 PMC5115610

[26] Bode DC Stanyon HF Hirani T Baker MD Nield J Viles JH. Serum albumin's protective inhibition of amyloid-β Fiber formation is suppressed by cholesterol, fatty acids and warfarin. J Mol Biol. (2018) 430:919–34. 10.1016/j.jmb.2018.01.008 29409811

[27] Llewellyn DJ Serum albumin concentration and cognitive impairment. Curr Alzheimer Res. (2009) 999:1–6. 10.2174/1872209197471563128 PMC2886725 20205675

[28] Costa M Horrillo R Ortiz AM Pérez A Mestre A Ruiz A. Increased albumin oxidation in cerebrospinal fluid and plasma from Alzheimer's disease patients. J Alzheimer Dis. (2018) 63:1395–404. 10.3233/JAD-180243 29782326 PMC6004933

[29] Chen R-L Chen CP-C Preston JE. Elevation of CSF albumin in old sheep: relations to CSF turnover and albumin extraction at blood-CSF barrier. J Neurochem. (2010) 113:1230–9. 10.1111/j.1471-4159.2010.06689.x 20236385

[30] Jin W-S Shen L-L Bu X-L Zhang W-W Chen S-H Huang Z-L. Peritoneal dialysis reduces amyloid-beta plasma levels in humans and attenuates Alzheimer-associated phenotypes in an APP/PS1 mouse model. Acta Neuropathol. (2017) 134:207–20. 10.1007/s00401-017-1721-y 28477083

[31] Kitaguchi N Hasegawa M Ito S Kawaguchi K Hiki Y Nakai S. A prospective study on blood Aβ levels and the cognitive function of patients with hemodialysis: a potential therapeutic strategy for Alzheimer's disease. J Neural Transm. (2015) 122:1593–607. 10.1007/s00702-015-1431-3 26228626

[32] Sakai K Senda T Hata R Kuroda M Hasegawa M Kato M. Patients that have Undergone Hemodialysis Exhibit Lower Amyloid Deposition in the Brain: evidence supporting a therapeutic strategy for Alzheimer's disease by removal of blood amyloid. J Alzheimers Dis. (2016) 51:997–1002. 10.3233/JAD-151139 26923028

[33] Tholen S Schmaderer C Chmielewski S Förstl H Heemann U Baumann M. Reduction of amyloid-β plasma levels by hemodialysis: an anti-amyloid treatment strategy? J Alzheimers Dis. (2016) 50:791–6. 10.3233/JAD-150662 26682683

[34] The AMBAR study Grifols Grifols Available online at: https://www.grifols.com/en/the-ambar-study (accessed June, 2019).

[35] Boada M Ramos-Fernández E Guivernau B Muñoz FJ Costa M Ortiz AM. Treatment of Alzheimer disease using combination therapy with plasma exchange and haemapheresis with albumin and intravenous immunoglobulin: rationale and treatment approach of the AMBAR (Alzheimer Management By Albumin Replacement) study. Neurologia. (2016) 31:473–81. 10.1016/j.nrl.2014.02.003 25023458

[36] Boada M López O Núñez L Szczepiorkowski ZM Torres M Grifols C. Plasma exchange for Alzheimer's disease Management by Albumin Replacement (AMBAR) trial: study design and progress. Alzheimers Dement (N.Y). (2019) 5:61–69. 10.1016/j.trci.2019.01.001 30859122 PMC6395854

[37] Boada M Anaya F Ortiz P Olazarán J Shua-Haim JR Obisesan TO. Efficacy and safety of plasma exchange with 5% albumin to modify cerebrospinal fluid and plasma amyloid-β concentrations and cognition outcomes in Alzheimer's disease patients: a multicenter, randomized, controlled clinical trial. J Alzheimer's Dis. (2017) 56:129–43. 10.3233/JAD-160565 27911295 PMC5240541

[38] DeMattos RB Bales KR Cummins DJ Dodart JC Paul SM Holtzman DM Peripheral anti-Aβ antibody alters CNS and plasma Aβ clearance and decreases brain A b burden in a mouse model of Alzheimer's disease. Proc Natl Acad Sci USA. (2001) 98:8850–5. 11438712 10.1073/pnas.151261398 PMC37524

[39] Boada M Ortiz P Anaya F Hernández I Muñoz J Núñez L. Amyloid-targeted therapeutics in Alzheimer's disease: use of human albumin in plasma exchange as a novel approach for Abeta mobilization. Drug News Perspect. (2009) 22:325–39. 10.1358/dnp.2009.22.6.1395256 19771322

[40] Skillbäck T Delsing L Synnergren J Mattsson N Janelidze S Nägga K. CSF/serum albumin ratio in dementias: a cross-sectional study on 1861 patients. Neurobiol Aging. (2017) 59:1–9. 10.1016/j.neurobiolaging.2017.06.028 28779628

[41] Shibata M Yamada S Kumar SR Calero M Bading J Frangione B. Clearance of Alzheimer's amyloid-ss(1-40) peptide from brain by LDL receptor-related protein-1 at the blood-brain barrier. J Clin Invest. (2000) 106:1489–99. 10.1172/JCI10498 11120756 PMC387254

[42] Reese LC Zhang W Dineley KT Kayed R Taglialatela G. Selective induction of calcineurin activity and signaling by oligomeric amyloid beta. Aging Cell. (2008) 7:824–35. 10.1111/j.1474-9726.2008.00434.x 18782350 PMC2954114

[43] Demuro A Mina E Kayed R Milton SC Parker I Glabe CG. Calcium dysregulation and membrane disruption as a ubiquitous neurotoxic mechanism of soluble amyloid oligomers. J Biol Chem. (2005) 280:17294–300. 10.1074/jbc.M500997200 15722360

[44] Felice FGD De Felice FG Vieira MNN Bomfim TR Decker H Velasco PT. Protection of synapses against Alzheimer's-linked toxins: Insulin signaling prevents the pathogenic binding of Aβ oligomers. Proc Natl Acad Sci USA. (2009) 106:1971–6. 10.1073/pnas.0809158106 19188609 PMC2634809

[45] Ferreira ST Lourenco MV Oliveira MM De Felice FG Soluble amyloid-Î^2^ oligomers as synaptotoxins leading to cognitive impairment in Alzheimer'disease. Front Cell Neurosci. 9:191 10.3389/fncel.2015.00191 26074767 PMC4443025

[46] Tomiyama T Matsuyama S Iso H Umeda T Takuma H Ohnishi K. A mouse model of amyloid oligomers: their contribution to synaptic alteration, abnormal tau phosphorylation, glial activation, and neuronal loss *in vivo*. J Neurosci. (2010) 30:4845–56. 10.1523/JNEUROSCI.5825-09.2010 20371804 PMC6632783

[47] Vega L Arroyo ÁA Tabernero A Medina JM. Albumin-blunted deleterious effect of amyloid-β by preventing the internalization of the peptide into neurons. J Alzheimers Dis. (2009) 17:795–805. 10.3233/JAD-2009-1093 19542622

[48] Ezra A Rabinovich-Nikitin I Rabinovich-Toidman P Solomon B. Multifunctional effect of human serum albumin reduces Alzheimer's disease related pathologies in the 3xTg mouse model. J Alzheimers Dis. (2016) 50:175–88. 10.3233/JAD-150694 26682687

[49] Patel SU Sadler PJ Tucker A Viles JH Direct detection of albumin in human blood plasma by proton NMR spectroscopy. Complexation of nickel2+. J Am Chem Soc. (1993) 115:9285–6. 10.1021/ja00073a053

[50] Sarell CJ Wilkinson SR Viles JH. Substoichiometric levels of Cu2+ ions accelerate the kinetics of fiber formation and promote cell toxicity of amyloid-β from Alzheimer disease. J Biol Chem. (2010) 285:41533–40. 10.1074/jbc.M110.171355 20974842 PMC3009880

[51] You H Tsutsui S Hameed S Kannanayakal TJ Chen L Xia P. Aβ neurotoxicity depends on interactions between copper ions, prion protein, and N-methyl-D-aspartate receptors. Proc Natl Acad Sci USA. (2012) 109:1737–42. 10.1073/pnas.1110789109 22307640 PMC3277185

[52] Viles JH Metal ions and amyloid fiber formation in neurodegenerative diseases. Copper, zinc and iron in Alzheimer's, Parkinson's and prion diseases. Coord Chem Rev. (2012) 256:2271–84. 10.1016/j.ccr.2012.05.003

[53] Shuvaev VV Laffont I Serot JM Fujii J Taniguchi N Siest G. Increased protein glycation in cerebrospinal fluid of Alzheimer's disease. Neurobiol Aging. (2001) 22:397–402. 10.1016/S0197-4580(00)00253-0 11378244

[54] Bakris GL Bank AJ Kass DA Neutel JM Preston RA Oparil S. Advanced glycation end-product cross-link breakers: a novel approach to cardiovascular pathologies related to the aging process. Am J Hypertens. (2004) 17:23S−30S. 10.1016/j.amjhyper.2004.08.022 15607432

[55] Schleicher E Wieland OH. Kinetic analysis of glycation as a tool for assessing the half-life of proteins. Biochim Biophys Acta. (1986) 884:199–205. 10.1016/0304-4165(86)90244-8 3768412

[56] Williams SK Devenny JJ Bitensky MW. Micropinocytic ingestion of glycosylated albumin by isolated microvessels: possible role in pathogenesis of diabetic microangiopathy. Proc Natl Acad Sci USA. (1981) 78:2393–7. 10.1073/pnas.78.4.2393 6941299 PMC319352

[57] Shackleton B Crawford F Bachmeier C. Inhibition of ADAM10 promotes the clearance of Aβ across the BBB by reducing LRP1 ectodomain shedding. Fluids Barriers CNS 13:14. 10.1186/s12987-016-0038-x 27503326 PMC4977753

[58] Wang H Chen F Zhong KL Tang SS Hu M Long Y PPARγ agonists regulate bidirectional transport of amyloid-β across the blood-brain barrier and hippocampus plasticity indb/dbmice. Brit J Pharmacol. (2016) 173:372–85. 10.1111/bph.13378 26507867 PMC5341223

[59] Donahue JE Flaherty SL Johanson CE Duncan JA Silverberg GD Miller MC. RAGE, LRP-1, and amyloid-beta protein in Alzheimer's disease. Acta Neuropathol. (2006) 112:405–15. 10.1007/s00401-006-0115-3 16865397

[60] Roberts KF Elbert DL Kasten TP Patterson BW Sigurdson WC Connors RE. Amyloid-β efflux from the central nervous system into the plasma. Ann Neurol. (2014) 76:837–44. 10.1002/ana.24270 25205593 PMC4355962

[61] Hladky SB Barrand MA. Elimination of substances from the brain parenchyma: efflux via perivascular pathways and via the blood-brain barrier. Fluids Barriers CNS. (2018) 15:30. 10.1186/s12987-018-0113-6 30340614 PMC6194691

[62] Iliff JJ Wang M Liao Y Plogg BA Peng W Gundersen GA. A paravascular pathway facilitates CSF flow through the brain parenchyma and the clearance of interstitial solutes, including amyloid β. Sci Transl Med. (2012) 4:147ra111. 10.1126/scitranslmed.3003748 22896675 PMC3551275

[63] Menéndez González M Implantable systems for continuous liquorpheresis and CSF replacement. Cureus. (2017) 9:e1022 10.7759/cureus.1022 28413734 PMC5388363

[64] Pascale CL Miller MC Chiu C Boylan M Caralopoulos IN Gonzalez L. Amyloid-beta transporter expression at the blood-CSF barrier is age-dependent. Fluids Barriers CNS. (2011) 8:21. 10.1186/2045-8118-8-21 21740544 PMC3162580

[65] Mawuenyega KG Sigurdson W Ovod V Munsell L Kasten T Morris JC. Decreased clearance of CNS -amyloid in Alzheimer's disease. Science. (2010) 330:1774. 10.1126/science.1197623 21148344 PMC3073454

